# When rehabilitation is not enough, how targeting metabolism can overcome the limited plasticity of skeletal muscle after traumatic injury

**DOI:** 10.1113/EP093074

**Published:** 2025-11-20

**Authors:** Sarah M. Greising, Jarrod A. Call

**Affiliations:** ^1^ School of Kinesiology University of Minnesota Minneapolis Minnesota USA; ^2^ Department of Physiology and Pharmacology University of Georgia Athens Georgia USA; ^3^ Regenerative Bioscience Center University of Georgia Athens Georgia USA

**Keywords:** β_2_‐adrenergic receptor agonist, neuromusculoskeletal injury, rehabilitation, skeletal muscle injury

## Abstract

There is a category of large‐scale neuromusculoskeletal injuries that result in long‐term functional disabilities, and one such injury is volumetric muscle loss (VML) injury. In addition to the clinical outcomes related to long‐term dysfunction, co‐morbidities and reduced mobility and physical activity, this review addresses several underexplored physiological limitations of VML at both the whole‐body level and within the injured muscle. Our objectives with this review are to provide: (1) critical understanding of the pathophysiological limitations related to VML injury; (2) evidence for emerging treatment options that target the VML‐induced metabolic dysregulation; and (3) early functional data on metabolic treatments as a component of regenerative rehabilitation. We highlight new attempts to intervene in this unique pathophysiology, in addition to current unanswered questions for the field.

## Abstract (highlights)

- **What is the topic of this review?**
  We aim to provide to the field a clear understanding of the sequela and physiological impact of the skeletal muscle and whole‐body metabolism following volumetric muscle loss injury.
- **What advances does it highlight?**
  Here, we add to the scientific conversation by bringing in an innovative and additive approach to treat metabolic impairments after injury in a regenerative rehabilitation programme. Although questions remain, this review provides a translational foundation to consider as the field grows.

## 1 INTRODUCTION

Skeletal muscle injuries refer to any insult that causes myofibre damage and can arise from various mechanisms, each influencing the recovery and repair processes. Mild skeletal muscle injuries, such as muscle strains, are a common occurrence, primarily resulting from unaccustomed high‐force contractions that negatively affect the ability of the muscle cell to regulate intracellular calcium release (i.e., excitation–contraction uncoupling). These types of muscle injuries typically have a recovery period, defined as the recovery of contractile strength, of 2–5 weeks (Greising et al., 2020; Willett et al., 2016).

Traumatic skeletal muscle injuries, such as volumetric muscle loss (VML) injury, are primarily attributable to either blunt force trauma, such as when an object strikes the body, or penetrating trauma, such as when an object pierces the body (Garg et al., 2025; Greising et al., 2020; Willett et al., 2016). These traumas cause direct damage to the skeletal myofibres and can also damage the innervating nerves, the perfusion vasculature and myotendinous junctions. Traumatic skeletal muscle injuries from blunt force or penetrating trauma can result secondary to any of the 150000 open fractures or 30000 gunshot wounds (trauma), 36000 chainsaw accidents (industrial/farm) and 13000 soft‐tissue sarcomas (cancer) that are taking place annually in the USA. They are also common among tactical athletes and active‐duty military members, owing to exposure to a high‐energy explosion or owing to a knife/bullet wound. The recovery of contractile strength after traumatic muscle injuries is protracted in comparison to mild muscle strains or might never fully recover in worst‐case scenarios, such as VML injuries.

Our recent work has provided evidence of a lesser‐known consequence of VML injury, metabolic dysregulation. Herein, we summarize recent advances in understanding skeletal muscle and whole‐body metabolic changes that accompany a VML injury, and we highlight successes and failures in attempting to intervene in this unique pathophysiology.

## 2 CRITICAL GAPS IN OUR UNDERSTANDING

### 2.1 Pathophysiology

**FIGURE 1:**
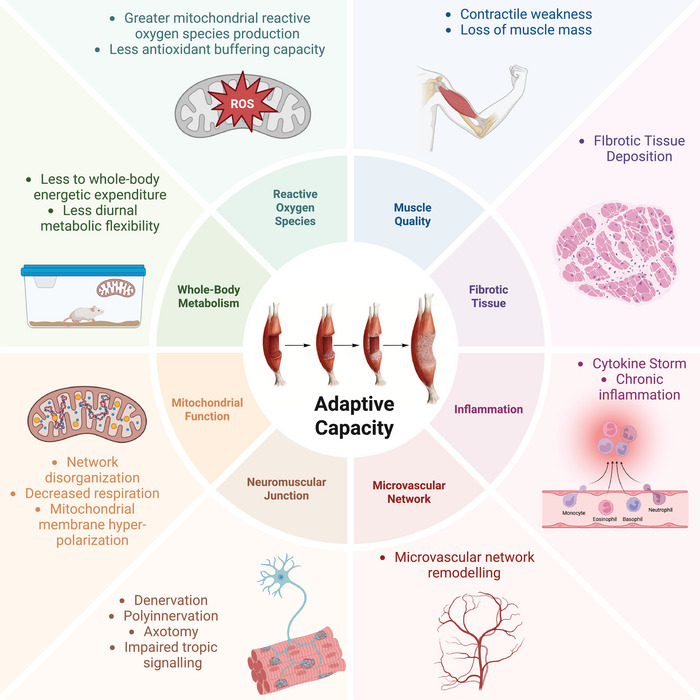
The injury sequelae after volumetric muscle loss injury have vast impacts on the skeletal muscle and supportive physiological structures. Along with the chronic lack of functional recovery, there are noteworthy limitations in the adaptive capacity of the muscle remaining after injury, which will limit the efficacy of any treatment approaches. Figure created in BioRender Call, J. (2025).

VML injury is most frequently characterized by chronic inflammation, the replacement of the lost contractile tissue with fibrotic tissue, a loss of functional vasculature, and denervation and/or changes in innervation patterns. Each VML injury is unique and therefore might not involve all the pathologies discussed below; however, a commonality among VML injuries is a lack of tissue regeneration that leads to an irrecoverable loss of muscle mass and contractile strength (Figure 1).

Regenerative and repair processes active following a VML injury are taking place in complex inflammatory conditions. VML‐injured muscles demonstrate a heightened and prolonged expression pattern of chemotactic, inflammatory and immune cell infiltration gene transcripts (Greising et al., 2016, 2017, 2018). Mild muscle injuries, such as muscle strains, experience a decrease in inflammatory signalling a few days after injury, whereas VML has been shown to maintain this sustained expression over ≥28 days postinjury (Aguilar et al., 2018).

The sustained inflammatory phase has been associated with complement activation of fibrosis‐inducing pathways, such as transforming growth factor‐β1 (TGF‐β1) and Wnt, that negatively affect the ability of the VML‐injured muscle to regulate the extracellular matrix. Persistent activation of TGF‐β1 and Wnt signalling drives fibro‐adipogenic precursor activity, increasing fibrosis and irregular extracellular matrix remodelling (Greising et al., 2017; Hoffman et al., 2022; Rodriguez et al., 2021). Impaired extracellular matrix remodelling suppresses muscle stem cell (i.e., satellite cell) activation, further blocking the ability of the cell to self‐regenerate. This results in an accumulation of fibrotic tissue that can chronically secrete cytokines and create a self‐perpetuating cycle of inflammation, fibrotic tissue remodelling and impaired muscle recovery.

The trauma that induces a VML injury might also damage the vasculature of the remaining skeletal muscle tissue. The total surface area of the vasculature and adequate perfusion of that surface area is crucial for maintaining the contractile and metabolic function of skeletal muscle, because the perfused vasculature of skeletal muscle supports essential exchange of gases, nutrients and waste and must adapt to large increases in metabolic demand during activity. Interestingly, current evidence suggests a compensatory increase in capillary density after a VML injury (Anderson et al., 2019; Southern et al., 2019), but the extent to which those capillaries are capable of adequate perfusion remains unclear.

Until recently, the pathology of innervation following VML injuries had not been examined thoroughly. Initial evidence of acetylcholine receptor clustering, a marker of neuromuscular junctions present throughout the muscle, suggested that neuromuscular junctions were either damaged or removed because of the VML injury (Anderson et al., 2019), and neuromuscular junction removal was supported by a separate analysis of nerve axotomy after VML (Corona et al., 2018). A more nuanced story emerged with two‐ and three‐dimensional reconstructions of the neuromuscular junctions along a time course of recovery. This work showed an expanded, yet highly fragmented, acetylcholine receptor area that was associated with ∼50% of myofibres being fully denervated or partly denervated acutely, and the proportion of partly denervated myofibres persisted chronically (Hoffman et al., 2023).

The strides the field has made in understanding VML pathophysiology have undoubtedly led to innovative experimental interventions leveraging this knowledge to improve muscle outcomes. Unfortunately, full functional recovery remains elusive. Our position is that failure to address the underlying pathophysiology undermines interventions from the start, particularly rehabilitation that relies on the endogenous cellular environment to accomplish the goals of myofibre adaptation (Figure 1).

### 2.2 Lack of adaptive capacity to physical rehabilitation

Regularly scheduled physical activity (i.e., exercise training) has a potent and pleiotropic effect on skeletal muscle attributes of contractile strength, metabolic function and muscle mass, because healthy, uninjured skeletal muscle readily adapts to mechanical and/or chemical cues to improve functional capacity. The foundation of effective evidence‐based rehabilitation approaches is that the muscle being treated maintains an adaptive capacity and will exhibit beneficial (in terms of functionality) remodelling because of an intervention. However, physical rehabilitation protocols, at least those tested experimentally to date, have not yet been considered a viable solution to correct skeletal muscle dysfunction after a VML injury when used in isolation or combined with other adjunctive interventions.

The only clinical trial of VML‐injured patients described a cohort of 13 patients, 7–120 months removed from the time of injury, who were treated initially with extensive physical therapy and still had significant functional deficits remaining (Dziki et al., 2016). In preclinical animal studies of rehabilitation, the data are far more robust, but unfortunately, study outcomes indicate that interventions are equally ineffective at correcting the VML pathophysiology. This includes studies that have tested physical rehabilitation including voluntary wheel running, chronic intermittent electrical stimulation, vibration and passive range of motion (Aurora et al., 2014; Basten et al., 2023; Habing et al., 2024; Hoffman et al., 2025; Hu et al., 2022; Johnson et al., 2024; McFaline‐Figueroa et al., 2025; Nicholson et al., 2024; Schifino et al., 2023; Southern et al., 2019; Ziemkiewicz et al., 2023).

Within our collective laboratories, we have been pursuing potential causes of the limited adaptive capacity for physical rehabilitation of VML‐injured muscle. One under‐investigated area that has particularly intrigued us is the extent to which the VML injury and subsequent pathophysiology noted above disrupts mitochondrial bioenergetics within the remaining skeletal myofibres. Below, we provide evidence of widespread changes to skeletal muscle and whole‐body metabolism following a VML injury and evidence that correcting attributes of the mitochondrial bioenergetics alone can lead to improved functionality and, potentially, better adaptive capacity.

### 2.3 VML affects muscle bioenergetics

**FIGURE 2:**
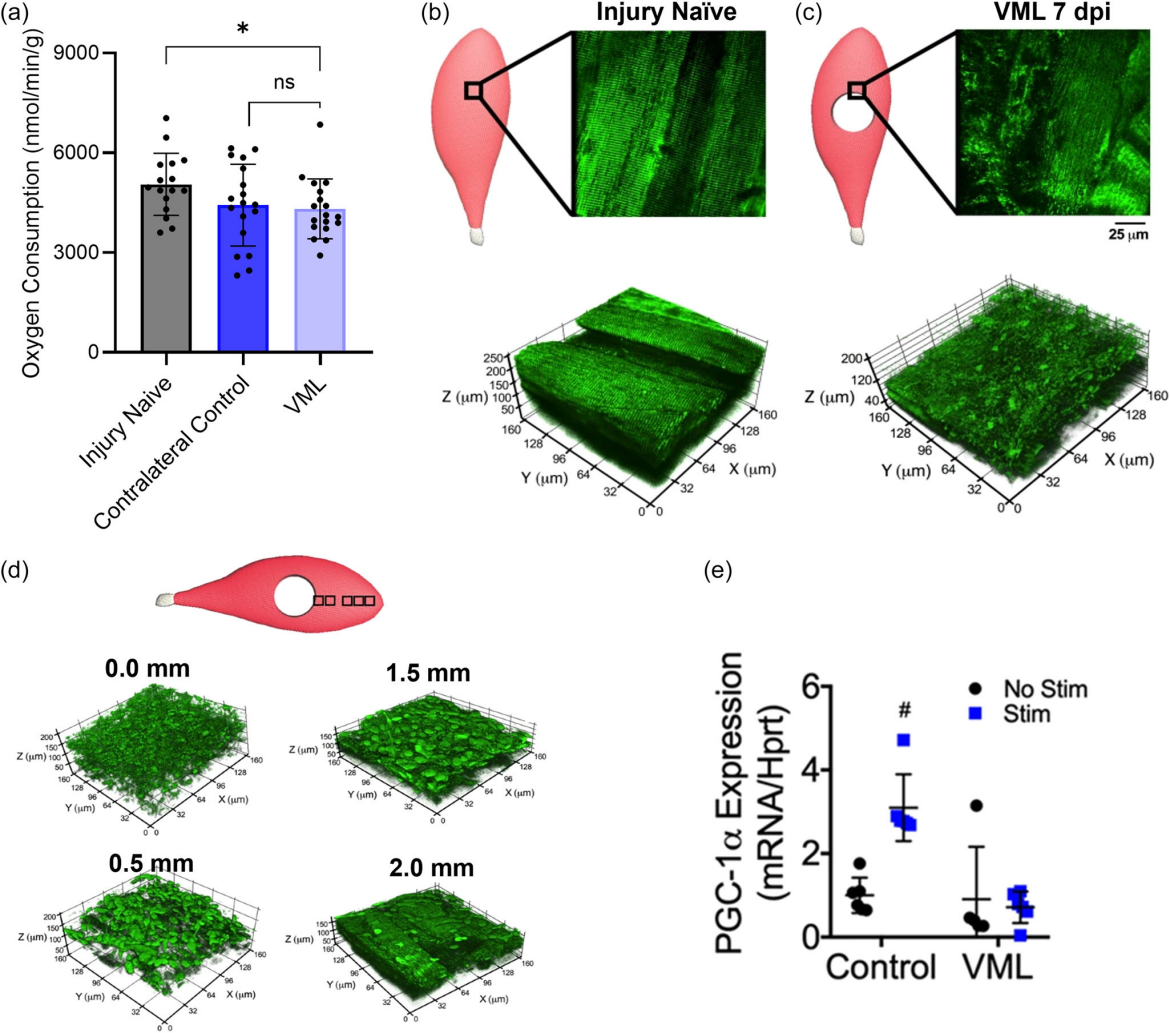
Changes in mitochondrial respiration, network organization and transcriptional regulation after volumetric muscle loss (VML) injury. (a) Oxygen consumption rates for permeabilized myofibres from completely uninjured animals (Injury Naïve), and permeabilized myofibres from VML injured animals, the contralateral uninjured limb (Contralateral Control) and injured limb (VML). *Significant difference (*p *< 0.05); ns, not significant. (b, c) Schematic diagrams representing injury‐naïve and VML‐injured myofibres at 7 days post‐injury (dpi), showing the region of interest evaluated during imaging and representative two‐dimensional images from the respective regions from transgenic mice that ubiquitously expressed mitochondrial Dendra2 green fluorescent protein. Representative three‐dimensional reconstruction of mitochondrial network adjacent to the VML injury site from injury‐naïve and VML‐injured myofibres. (d) Schematic diagram showing an injured myofibre with boxes to indicate the imaging sites at increasing distances from the border of the VML injury towards the origin of the muscle. Representative images from 0, 0.5, 1.5 and 2.0 mm away from the proximal VML injury border. The reader is directed to Southern et al. (2019) for specific details and results on the mitochondrial network analysis. (e) *PGC‐1α* gene expression 3 h after completion of stimulation protocol (30 min of stimulation) for stimulated and non‐stimulated limbs of control and VML‐injured mice; two‐way ANOVA, interaction *p* < 0.001, ^#^Significantly different from all other experimental groups. Image reproduced from Greising et al. (2018); Southern et al. (2019) under Open Access Creative Commons CC‐BY license; readers are pointed to the primary papers for experimental specifics.

In 2018, we were the first to report that a decrease in mitochondrial respiration (i.e., oxygen consumption) was an attribute of VML injury (Greising et al., 2018). This preclinical study included a time course analysis of contractile and metabolic function (1, 2 and 4 months postinjury) of VML‐injured muscles with and without intervention. Then, and now, we focused our examination on mitochondrial respiration in permeabilized myofibres isolated from the remaining muscle after a VML injury, at the border of the defect, using myofibre bundles that did not show signs of tissue damage from the injury. We decided on permeabilized myofibres to capture mitochondrial function in the context of an intact mitochondrial network as opposed to isolated mitochondrial fractions. A very important lesson that we learned from this first study is that moving forwards we needed to use permeabilized myofibres from a completely uninjured control (injury‐naïve mouse) instead of the contralateral control limb. Our statistical analysis of mitochondrial respiration did not show a difference between injured and uninjured limbs within a VML‐injured animal; however, respiration was 25% less when compared with an injury‐naïve animal (Figure 2a). In retrospect, this was the first indication that the VML injury might have systemic metabolic effects, a topic covered in more depth in the whole‐body metabolism section below (section 2.6).

The most robust skeletal muscle adaptation to endurance exercise training is an increase in oxidative capacity. Naturally, we decided to investigate whether a voluntary wheel‐running intervention could rescue mitochondrial respiration in permeabilized myofibres from VML‐injured muscle (Southern et al., 2019). The mitochondrial respiration rates increased with voluntary wheel running in injury‐naïve mice, whereas there were no statistically significant differences in VML‐injured animals with or without exercise intervention. This study included two complementary outcomes that shed light on why mitochondrial respiration is reduced with VML injury and why VML‐injured muscle did not show an adaptive capacity to voluntary wheel running.

First, using multi‐photon microscopy and a mouse that expresses a green fluorescent protein tag on all mitochondria (Figure 2b,c), we rigorously investigated the mitochondrial network within the remaining muscle after a VML injury across time and at different distances from the injury (Figure 2d). There was an early decrease in mitochondria network organization that persisted until ≥1 month postinjury. Furthermore, the mitochondrial network was disorganized throughout 20% of the muscle length away from the site of the VML injury. Mitochondria bioenergetic efficiency is supported by a highly connected mitochondrial network; therefore, the changes in network organization are likely to contribute to the reduced mitochondrial respiration.

The second striking finding from this study is that the transcription factor peroxisome proliferator‐activated receptor gamma coactivator 1‐alpha (PGC1α), which facilitates mitochondrial gene regulation, was less sensitive to acute activation in VML‐injured myofibres relative to myofibres from injury‐naïve mice. An acute electrical stimulation protocol produced a 3‐fold increase in *PGC1α* mRNA expression in injury‐naïve animals and had no effect on VML‐injured myofibres (Figure 2e). The inability to engage mitochondrial transcription factors, such as PGC1α, would certainly contribute to a lack of adaptive capacity with voluntary wheel running.

On this point, our mitochondrial respiration analysis of VML‐injured permeabilized myofibres was rather simplistic, in that the approach used a supraphysiological concentration of ADP and a bolus exposure of malate, glutamate and succinate to stimulate respiration. We considered that these conditions might over‐exaggerate or even mask attributes of mitochondrial function. Notably, our analysis also did not include a marker of mitochondrial membrane potential.

**FIGURE 3:**
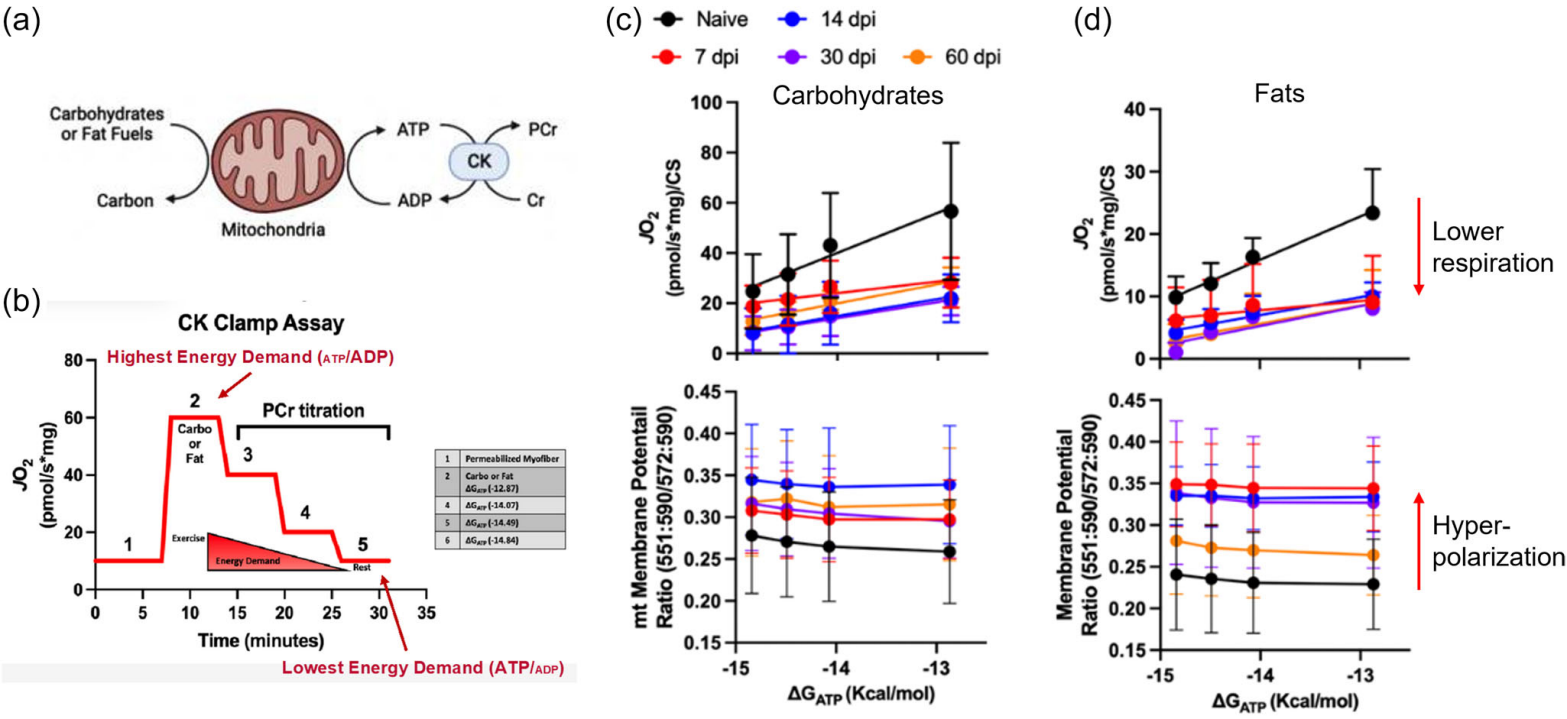
Changes in permeabilized myofibre respiration (*J*O_2_) and mitochondrial membrane potential across clamped energetic demands after volumetric muscle loss (VML) injury. (a) Conceptual framework for creatine kinase (CK) clamp technique and mitochondrial membrane potential evaluation. CK catalyses the reversible reaction of creatine (Cr) and ATP to produce phosphocreatine (PCr) and ADP. (b) During the CK clamp test, the ATP resynthesis demand (∆*G*_ATP_) can be manipulated by titrating PCr and has the effect of decreasing *J*O_2_. (c) The relationship between ∆*G*_ATP_ and *J*O_2_ (top) and mitochondrial membrane potential (bottom) using carbohydrate substrates at different times after VML injury. (d) The relationship between ∆*G*_ATP_ and *J*O_2_ (top) and mitochondrial membrane potential (bottom) using fat substrates at different times after VML injury. Abbreviation: dpi, days postinjury. Image reproduced from Heo et al. (2025) under Open Access Creative Commons CC‐BY‐NC license; readers are pointed to the primary paper for experimental specifics.

These concerns were addressed directly across two studies published in 2023 using a ‘CK clamp’ technique that leverages the enzymatic reaction of creatine kinase (CK) and phosphocreatine to stepwise ‘clamp’ ATP/ADP ratios at different ATP resynthesis demands (Figure 3a,b) (Heo et al., 2023; McFaline‐Figueroa et al., 2023). Mitochondrial respiration and simultaneous mitochondrial membrane potential were evaluated using the CK clamp technique under carbohydrate‐ or fat‐supported substrate influence (pyruvate/malate/succinate vs. palmitoyl‐carnitine/malate, respectively; Figure 3c,d). Collectively, these studies provide strong evidence for a profound consequence of VML injury on mitochondrial function as early as 3 days postinjury and extending to 2 months postinjury. Briefly, mitochondria from VML‐injured limbs have a reduced capacity to ramp respiration up or down based on ATP demand, and this coincides with a hyperpolarized (relatively more negative) mitochondrial membrane potential, suggesting that the proton motive force established by oxygen consumption is not being used efficiently to generate ATP.

When the mitochondrial membrane potential becomes hyperpolarized alongside a diminished capacity for ATP resynthesis, the resulting proton motive force equilibrates with an elevated redox state (e.g., high NADH to NAD^+^ ratio). This shift raises the reduction potential of multiple redox centres along the electron transport chain (e.g., complex I, complex III), increasing the likelihood of electron leakage to oxygen and thereby promoting generation of reactive oxygen species (ROS). Hence, oxidative stress is emerging as a cellular mechanism that might link VML‐induced mitochondrial dysfunction to the reduced adaptive capacity of the remaining myofibres.

**FIGURE 4:**
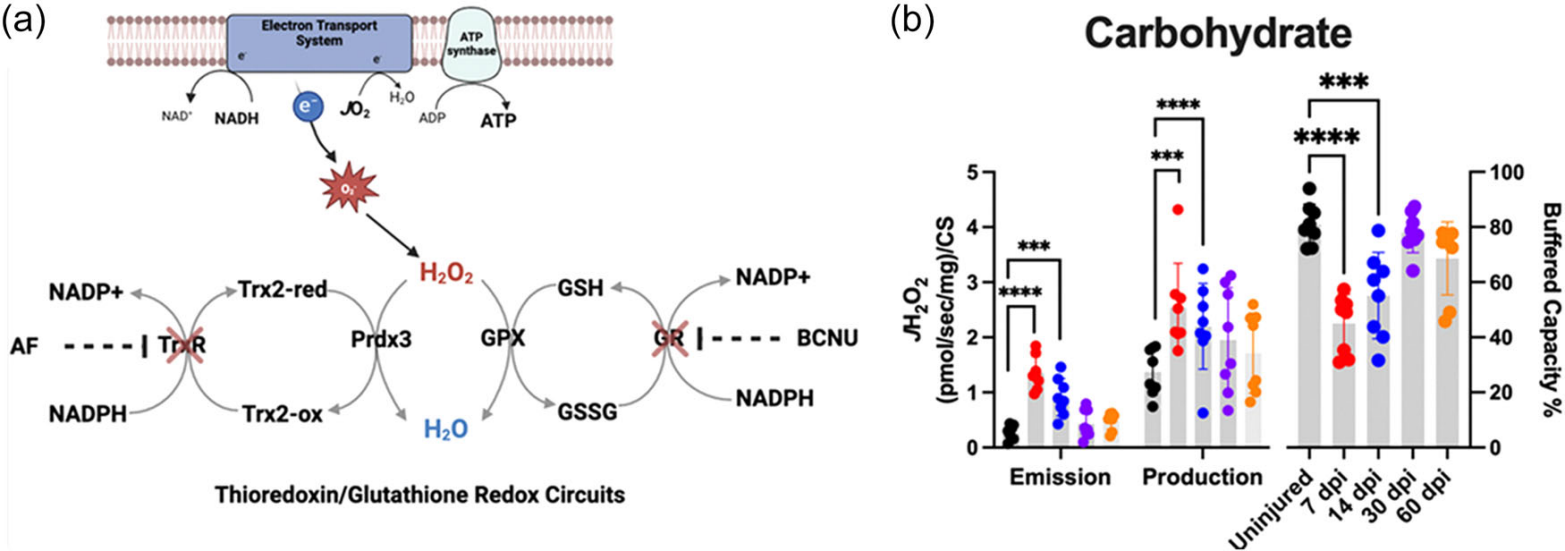
Time course of changes in volumetric muscle loss (VML)‐injured permeabilized myofibre reactive oxygen species emission, production and antioxidant buffering capacity. (a) Schematic diagram of endogenous H_2_O_2_ buffering circuits within mitochondria with inhibitors against those circuits indicated. (b) Reactive oxygen species emission, production and antioxidant buffer capacity for carbohydrate. Statistical significance was determined by one‐way ANOVA with Tukey's HSD *post hoc* test. Data are expressed as the mean ± SD. ***p < 0.001 and ****p < 0.0001. Abbreviation: dpi, days postinjury. Image reproduced from Heo et al. (2025) under Open Access Creative Commons CC‐BY‐NC license; readers are pointed to the primary paper for experimental specifics.

The final featured study of mitochondrial function from our group directly tested the hypothesis that VML‐induced mitochondrial hyperpolarization leads to greater ROS production that overwhelms the antioxidant buffering systems and contributes to mitochondrial dysfunction (Heo et al., 2025). Mitochondrial ROS emission (defined as ROS not quenched by the antioxidant buffering systems) and ROS production (defined as mitochondrial ROS generated and not quenched by the thioredoxin and glutathione reductase antioxidant systems) were investigated, and both were found to be elevated during the first 2 weeks postinjury (Figure 4a,b), with the primary leak sites identified as complex I_Q_, complex II_F_ and complex III_Qo_. The investigation into ROS emission and production provided robust evidence that mitochondrial hyperpolarization was associated with altered redox balance within the remaining muscle after VML injury. This work also supported the concept that excessive ROS emission after injury, particularly during the first 2 weeks postinjury, causes changes in the abundances of mitochondrially related proteins and negatively affects cellular bioenergetics. The extent to which this cellular environment contributes to the reticent adaptive capability after VML injury is currently being investigated by our group.

### 2.4 Effect of VML on mitochondrial enzyme activities

The proton motive force that provides the potential energy to drive ATP resynthesis is established across the inner mitochondrial membrane through the proton pumping of complex I, complex III and complex IV. These complexes pump protons against a concentration gradient, and the energy to accomplish this task is provided by the redox reactions of the electron transport system. Specifically, as the electrons that are removed from NADH and FADH_2_ (at complex I and complex II, respectively) are pulled through the electron transport system by oxygen, nearly 1 V of reduction potential is generated to power proton movement across the inner mitochondrial membrane. NADH and FADH_2_ accumulate from dehydrogenases in Krebs cycle, glycolysis and β‐oxidation that remove electrons from carbon‐based fuel stores (e.g., glycogen, fatty acids).

Through several studies, we have examined the extent to which the VML injury affects mitochondrial enzyme activities that could contribute to the altered bioenergetic efficiency. The most frequently tested enzyme is citrate synthase (CS), which serves as both a rate‐limiting enzyme of the Krebs cycle and an established indirect marker of mitochondrial content in skeletal muscle (Larsen et al., 2012). Despite the changes in mitochondrial network organization and mitochondrial bioenergetics, there is little evidence that VML injury affects CS enzyme activity. In fact, across the eight studies in which we have assessed CS enzyme activity (Bruzina et al., 2024; Corona et al., 2018; Dalske et al., 2021; Heo et al., 2025, 2023; McFaline‐Figueroa et al., 2025; Raymond‐Pope et al., 2023; Southern et al., 2019), there has been a significant difference between VML and injury‐naïve muscles only once, and it was a greater CS activity at 21 days postinjury only (Corona et al., 2018). This leads us to consider that the bioenergetic inefficiency with VML injury is not attributable to a decrease in mitochondrial content and is, instead, a consequence of the disrupted network organization and ROS‐induced changes to the mitochondrial proteins.

Other mitochondrial enzyme activities that we have explored include those contributing to fatty‐acid oxidation (3‐hydroxyacyl‐CoA dehydrogenase) (Bruzina et al., 2024; Raymond‐Pope et al., 2023), the Krebs cycle (alpha‐ ketoglutarate dehydrogenase, malate dehydrogenase and succinate dehydrogenase) (Dalske et al., 2021; Heo et al., 2023), pyruvate oxidation (pyruvate dehydrogenase) (Heo et al., 2023; McFaline‐Figueroa et al., 2025) and the electron transport system (complex I, complex II) (Dalske et al., 2021; Heo et al., 2023; McFaline‐Figueroa et al., 2025). The strength of evidence for whether these mitochondrial activities are affected by VML injury is weaker owing to being analysed in only a single study or contrasting results. For example, in two different studies we detected a decrease in 3‐hydroxyacyl‐CoA dehydrogenase activity (Bruzina et al., 2024) that could support changes in lipid oxidation, and yet in the other study there was no significant difference in 3‐hydroxyacyl‐CoA dehydrogenase activity when compared with injury‐naïve animals (Raymond‐Pope et al., 2023).

### 2.5 Summary of the time course of skeletal muscle bioenergetic pathophysiology after VML

The studies summarized above help to establish a well‐supported time course of bioenergetic changes to skeletal muscle after a VML injury. Using the outcome of electron conductance that reflects the ability of permeabilized myofibres to change oxygen consumption to match ATP resynthesis demand, the percentage change from uninjured naïve myofibres can be summarized as follows: days after VML injury, 1 (−60%), 5 (−52%), 7 (−35%), 10 (−59%), 14 (−41%), 30 (−16%) and 60 (−21%) (Heo et al., 2025; McFaline‐Figueroa et al., 2023, 2025). Moreover, the early temporal reductions in electron conductance coincide with a greater rate of ROS production and less antioxidant buffering capacity up to 14 days postinjury (McFaline‐Figueroa et al., 2025). The physiological time course of bioenergetic responses to VML injury is supported by similar changes in the metabolic transcriptome. For example, a gene set enrichment analysis showed that there were 60, 68, 58 and 65 significantly downregulated mitochondrial gene sets at days 3, 7, 14 and 28 days after VML, respectively (Miller et al., 2025). Among the most significantly downregulated gene sets were those encoding for proteins associated with oxidative phosphorylation, complex I of the respiratory chain, oxidative phosphorylation assembly factors, and carbohydrate and fatty acid oxidation. What remains unresolved with regard to this time course of bioenergetic dysfunction after VML injury is the extent to which the mitochondrial proteome is affected and the mechanism by which changes to the mitochondrial proteome abundance or function contribute to the bioenergetics of VML‐injured myofibres.

### 2.6 VML affects whole‐body metabolism

We began to suspect that a traumatic injury, such as VML injury, could be associated with systemic changes in physiology after learning from our experience that the uninjured contralateral limb muscle was not a suitable internal control for mitochondrial respiration testing (Greising et al., 2018). At the time, the idea that a traumatic muscle injury could have whole‐body effects was supported by the persistent systemic inflammation associated with the VML injury and a retrospective analysis of Iranian veteran amputees with a high prevalence of metabolic syndrome (Ejtahed et al., 2017). However, the extent to which the VML injury specifically affected whole‐body metabolism was unclear.

**FIGURE 5:**
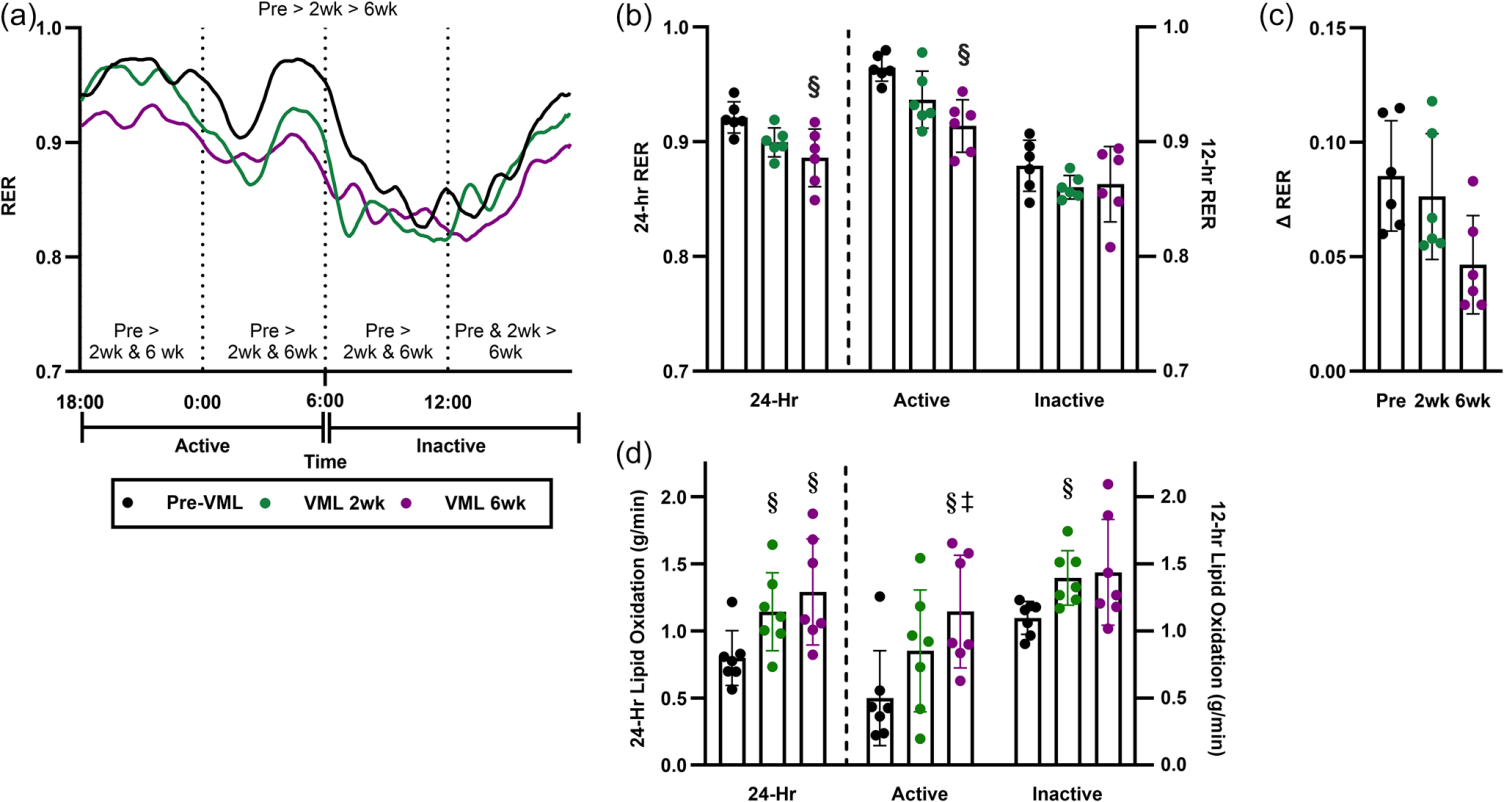
Time course of changes in whole‐body metabolism following volumetric muscle loss (VML) injury, 24 h data collected pre‐VML and 2 and 6 weeks post‐VML. (a, b) The respiratory exchange ratio (RER) across 24 h and broken down into 6 (dotted lines) and 12 h bins. In (b), the dashed line distinguishes the 24 h data and the two 12‐h activity and inactivity periods. VML progressively impairs metabolic flexibility. (c) The absolute delta (Δ)RER, as the difference between inactive and active periods. (d) Lipid oxidation increased over the 6 weeks post‐VML, mostly driven during the active period. Statistical significance was determined by one‐ and two‐way ANOVA with Tukey's HSD *post hoc* test. Data are expressed as the mean ± SD. different from: ^§^pre‐VML; ^‡^VML 2 weeks. Image reproduced from Dalske et al. (2021) under Open Access Creative Commons CC‐BY license; readers are pointed to the primary paper for experimental specifics.

To address this question directly, we carried out a longitudinal assessment of whole‐body metabolism, respiratory exchange ratio (RER), and carbohydrate and fat oxidation rates prior to a VML injury and at 2 and 6 weeks postinjury in adult mice (Dalske et al., 2021). A metabolic cage system was used to track ambulation, O_2_ consumption and CO_2_ production to assess whole‐body metabolism indirectly. There was a 10% decrease in whole‐body energy expenditure at 6 weeks post‐injury compared with pre‐injury (Figure 5a–c). The RER, ranging from 0.7 to 1.0, is used to determine the proportion of substrate used as fuel, with higher values indicating preference for carbohydrate oxidation and lower values indicating preference for lipid oxidation. The 24 h RER was 4% less at 6 weeks compared with pre‐injury, and this was supported by a decrease in carbohydrate oxidation and increase in lipid oxidation (Figure 5d) during the 6 week postinjury time course. Interestingly, these whole‐body metabolic changes had happened in the context of persevered ambulation, because the total distances covered in the cage for a 24 h period pre‐injury and 6 weeks postinjury were not different.

Traumatic muscle injuries are associated with reductions in physical activity; therefore, we wanted to explore whole‐body metabolism after VML injury further by creating models with limited ambulation. To do this, we created Plexiglass containers (12 cm×8.5 cm×6.3 cm) that fitted inside a standard cage and resulted in a ∼50% reduction in total daily ambulation. Across two studies published in 2023, we compared whole‐body metabolism in VML‐injured mice with restriction of physical activity versus VML‐injured mice without restriction of physical activity (Basten et al., 2023; Raymond‐Pope et al., 2023). The combination of VML injury and physical activity restriction resulted in a decrease in whole‐body energy expenditure and an increase in RER driven by reduced lipid oxidation when compared with VML‐injured mice with no physical activity restriction at 6 weeks after VML. These findings supported our initial findings that the time course of the VML pathophysiology is associated with whole‐body metabolic changes and that those whole‐body metabolic changes could be affected further by limited mobility after injury.

Our latest study into the effects of VML injury on whole‐body metabolism advanced the concept by exploring diurnal changes in RER (Bruzina et al., 2024). Diurnal RER, or the ability of the body to switch fuels throughout active and resting phases of day (e.g., metabolic flexibility) was analysed using a metabolic cage and separating the data into bins of the 12 h light (inactive) and 12 h dark (active) cycles. The VML‐injured mice had greater active phase RER compared with injury‐naïve mice, and this was driven by a decrease in lipid oxidation. An untargeted metabolomics analysis was used to determine whether these whole‐body changes were associated with the accumulation of certain metabolites in skeletal muscle. Several significant alterations in the metabolome occurred following VML injury. Notably, 93% of upregulated metabolites following VML were triglycerides containing long‐chain fatty acids, suggesting that VML injury might promote an adverse accumulation of localized triglycerides. This evidence agrees with the overall decrease in lipid oxidation at the whole‐body level (Raymond‐Pope et al., 2023) and with established deficiencies in lipid oxidation in VML‐injured permeabilized myofibres (McFaline‐Figueroa et al., 2023).

Whole‐body changes in energy expenditure and metabolic flexibility have been most widely reported in conditions of diabetes, obesity and bedrest, and there are also notable changes associated with traumas such as burn injuries. Our findings in the rodent VML model are supported clinically (Ejtahed et al., 2017; Tropf et al., 2023); however, the mechanisms for the whole‐body effects and the broader implications remain unclear.

## 3 EMERGING TREATMENT OPTIONS

Treatment options that address the lost skeletal muscle mass and function following VML have continued to develop over the past decades since VML was defined clinically (Grogan et al., 2011). Although outside of the scope of our review, there have been considerable advancements across the translational pipeline. There have been several recent review articles, to which the reader is directed, that go into significant detail on cell therapies, growth factors, tissue‐engineered strategies and their various combinations as treatment approaches (Corona & Greising, 2016; Garg et al., 2025; Saunders & Rose, 2021). Yet, to date only acellular bioengineered scaffolds have advanced to clinical options for the VML injured (Dziki et al., 2016). Physical rehabilitation continues to be a mainstay for the VML‐injured population, although physiological limitations in the response to rehabilitation remain unaddressed, as noted above. We continue to focus our interventions and emerging treatment targets that address the pathophysiological response to VML. To date, two options continue to drive innovation for the VML‐injured population within our laboratories: adjunctive pharmaceuticals [often repurposed US Food and Drug Administration (FDA)‐approved options] and regenerative rehabilitation, which may also be layered on the adjunctive pharmaceuticals.

The repurposing of clinical stage and FDA‐approved drugs is an approach that has long been supported by funding agencies (Saunders & Rose, 2021) to expedite clinical translation and provide options to the patients. From a regulatory standpoint, drug repurposing allows evaluation of pharmaceuticals with a known safety profile. Coupling this regulatory strategy with physiologically relevant skeletal muscle targets, anti‐fibrotic agents, immunomodulators and metabolism enhancers has been evaluated by us and others. Drug targeting for metabolism has been expanding rapidly, because therapeutics for diabetes and other associated metabolic disorders are now common. Driven in part by our combined research group, targeting of metabolism following VML injury has also continued to expand by targeting the PGC1α pathway via the β_2_‐adrenergic receptor agonist, formoterol, and mitochondrial ROS using antioxidant therapies.

Through a series of recent, complementary designed studies, we have established the foundational efficacy for adjunctive formoterol treatment to improve skeletal muscle mass and function following VML. Within its FDA‐approved use, formoterol is a component of bronchodilators, within long‐lasting inhalers for use in asthma or chronic obstructive pulmonary disease. Formoterol acts via a type of G‐protein‐coupled receptor, the β_2_‐andrenergic receptor, which is abundant in skeletal muscle. Formoterol is known to improve mitochondrial function through activation of PGC1α; in other off‐label‐type uses it is known to enhance skeletal muscle hypertrophy via the PI3K/Akt/mTOR pathway (Salazar‐Degracia et al., 2018). And it has been used in a handful of clinical investigations for other skeletal muscle indications (Lee et al., 2015, 2013). Collectively, the action of formoterol on skeletal muscle provides confidence for its use following VML injury. To establish efficacy, we focus on key metrics of skeletal muscle quality (e.g., mass and the number of myofibres) and function (i.e., maximal contractile ability and mitochondrial bioenergetics) (Bijwadia et al., 2023; McFaline‐Figueroa et al., 2025, 2022; Raymond‐Pope et al., 2023; Southern et al., 2019). To date, our studies have been for 1–2 months in adult male mice on a C57Bl/6 background, following the initial VML injury, and treatment (primarily oral formoterol) began immediately following injury and remained until terminal experiments.

Importantly, the initial foundational and mechanistic evaluation originated with an identification that we and others made, that with a known stimulus, such as exercise or rehabilitation (i.e., wheel running or electrical stimulation of muscle), the muscle remaining after VML injury does not activate pathways to upregulate gene expression of *PGC1α* (Southern et al., 2019), as described above. To overcome physiological pathways, we overexpressed *PGC1α* in the muscle immediately post‐VML injury, and 1 month post‐VML and transfection there was 25%–35% greater mitochondrial function (i.e., state 3 respiration) and mitochondrial content (i.e., CS activity) compared with control. These mitochondrial improvements were coupled with greater maximal torque production and contractile fatigue resistance in VML‐injured muscle with *PGC1α* overexpression. We used the work of Southern et al. (2019) as the overarching hypothesis to our ongoing translational work, that the muscle remaining after VML injury is physiologically limited and fails to induce mitochondrial biogenesis following rehabilitation. Furthermore, Southern's work identified a potential developmental target to restore mitochondrial biogenesis and, by extension, oxidative capacity and contractile function in the muscle remaining after VML injury. We targeted metabolism with overexpression of PGC1α initially, then formoterol in a drug‐repurposing approach providing an initial path to translation if successful.

**FIGURE 6:**
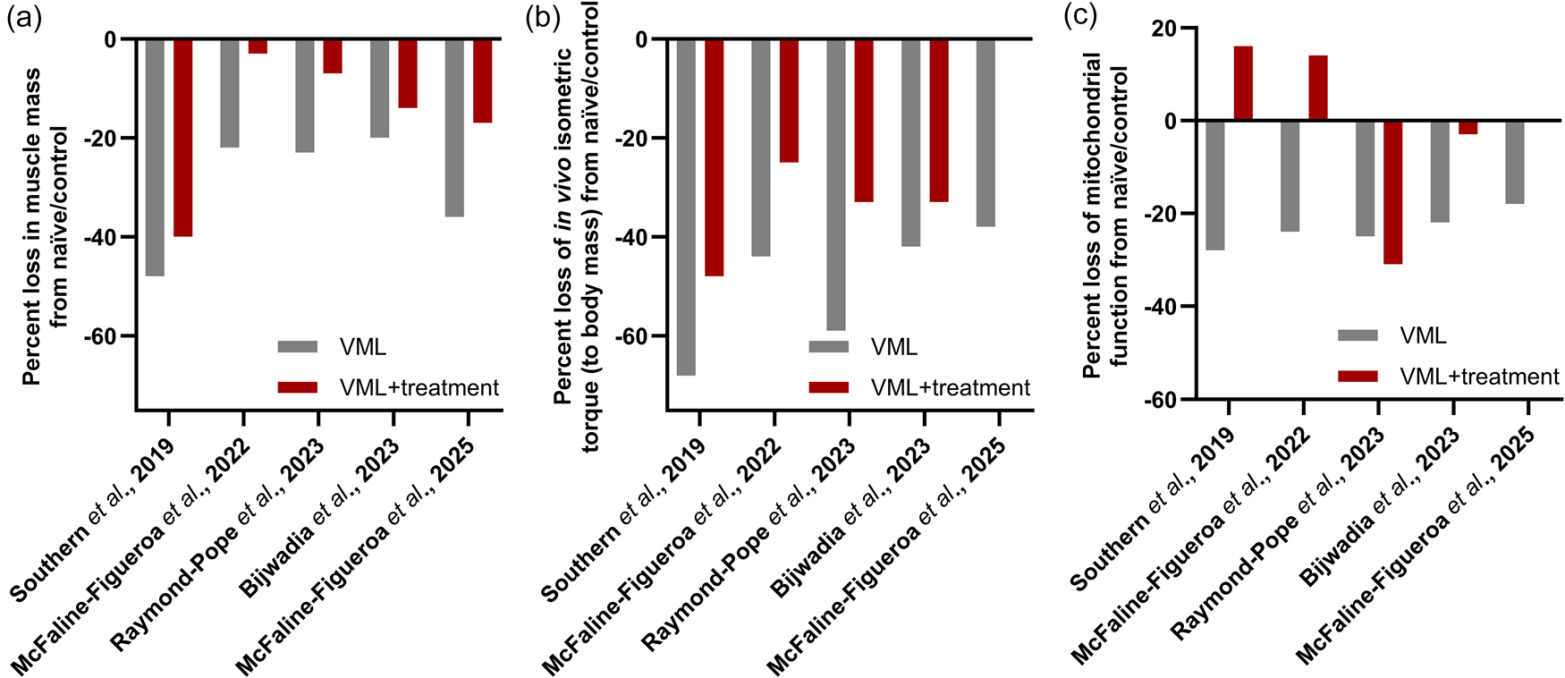
Across multiple studies with similar design, volumetric muscle loss (VML) injury results in loss of muscle mass (a) and function (b), while treatments (i.e., PGC1α or formoterol) at the time of injury, including overexpression of *PGC1α* and formoterol, are able to mitigate VML‐induced decline. (c) Mitochondrial function is captured within various data types, including peak oxygen consumption rate (ORC) during the creatine kinase (CK) clamp, mitochondrial respiratory function normalized to mitochondrial content, and 24 h whole‐body lipid oxidation. Note that, for a single paper (McFaline‐Figueroa et al., 2025), there was no torque or mitochondrial data collected for the formoterol treatment alone, and that was omitted in the figure. As needed, naïve/control data were used across papers. Data reproduced from Bijwadia et al. (2023); McFaline‐Figueroa et al. (2025, 2022); Raymond‐Pope et al. (2023); Southern et al. (2019) under various Open Access Creative Commons CC‐BY licenses; readers are pointed to the primary papers for experimental specifics.

A primary goal for treatment of skeletal muscle following VML is restoration of mass and structure (Grogan et al., 2011). With our consideration of metabolic bioenergetics, we believe that any treatment also needs to address the metabolism within the muscle. In the simplest aspect, muscle mass is a major component of muscle quality, along with the content of the muscle, be that protein, fibrotic and/or lipid components. Across our studies, formoterol treatment post‐VML has broadly supported increased muscle mass, although treatment alone has not been able to restore mass completely to pre‐morbid levels (Figure 6a). The composition of the muscle is also key to quality, including total/contractile protein content, which supports the contractile ability of the muscle, and the non‐contractile aspects, such as the extracellular matrix. To date, we have not identified an impact of either VML or formoterol on the protein content per unit of muscle. The composition of collagen in the muscle, in addition to the fibrotic accumulation, does not contribute to contractile ability of the muscle but does provide structural support and transmission of force. Fibrotic accumulation and collagen have a greater density than skeletal muscle, ∼1.3 g/cm^3^, in comparison to 1.06 g/cm^3^, hence it is important to understand how the composition of the muscle is changing. Using an indirect histological measure of collagen accumulation, formoterol treatment showed signs of reducing the overall burden on both densely and loosely packed collagen (McFaline‐Figueroa et al., 2025). Although not well characterized to date, lipid accumulation within the muscle after VML injury has been suggested anecdotally. We have not evaluated directly whether formoterol can limit lipid accumulation, but our data on whole‐body metabolism supports a possible benefit of treatment. Improved glucose tolerance with formoterol treatment following VML (Raymond‐Pope et al., 2023) suggests that formoterol might reduce fatty acid oversupply and support proper substrate partitioning, thereby limiting ectopic lipid deposition in the remaining muscle. Finally, cross‐sectional area and the total number of myofibres complement the quality measures of the muscle. Within an uninjured model, myofibre cross‐sectional area of the muscle was also increased after formoterol treatment (Bijwadia et al., 2023). Across our model, VML injury results in ∼30%–35% deficit of total myofibres in the mid‐belly of the muscle, and formoterol treatment improves that deficit to only ∼14%–17% (Bijwadia et al., 2023; McFaline‐Figueroa et al., 2025), suggesting that *de novo* regeneration of myofibres might be occurring, although more work is needed to draw a definite conclusion. Through our studies, there is strong support that formoterol is contributing to the recovery of the muscle following VML.

Although the restoration of pre‐morbid mass and structure is important, the ability to recover function of the muscle remaining after injury, specifically maximal contractile ability and mitochondrial function, is imperative to long‐term health and function. The goal of any skeletal muscle treatment needs to improve function, with the primary function of muscle being to contract and produce force. All our studies have included the evaluation of nerve‐stimulated maximal in vivo torque of the plantar‐ and/or dorsiflexor muscle groups (Figure 6b). VML injury alone results in a chronic non‐recoverable loss of torque, with deficits ranging from ∼38% to 68% in our studies. In our first attempt to overcome the VML pathology using overexpression of *PGC1α* at 1 month after injury, there was only a 10% functional deficit in torque compared with control, when accounting for muscle mass. Building on this success, the treatment with formoterol was evaluated following 2 months of treatment, with the majority of the studies indicating some mitigation of the functional deficit of injury, with only ∼25%–33% deficit remaining. Spanning the vast characterization of the muscle bioenergetics and mitochondrial enzyme activities, formoterol treatment continues to indicate robust effects after VML (Figure 6c). Although the studies have a range of methodological approaches, our initial evaluations focused on mitochondrial respiratory function normalized to mitochondrial content, and overexpression of *PGC1α* resulted in improvements above pre‐morbid levels (Southern et al., 2019). A similar magnitude of improvement was determined with formoterol treatment (McFaline‐Figueroa et al., 2022). Although not all our recent evaluations have included measures of whole‐body metabolism, we do have evidence that formoterol treatment post‐VML can recover VML‐induced impairments in lipid oxidation (Bijwadia et al., 2023). Furthermore, when challenged with a reduced ambulatory environment, as described above, formoterol treatment was able to mitigate some of the VML‐induced metabolic inflexibility (Raymond‐Pope et al., 2023). With the recovery of muscle mass and function combined with improvements in local and whole‐body metabolic function, formoterol continues to show promise for use following VML injury.

We recently expanded our metabolic targeting to mitochondrial ROS using antioxidant therapies (Heo et al., 2025). As noted above, following VML injury the ROS emission determined at the mitochondria increases temporarily after injury. Thus, to link ROS emissions more definitely to mitochondrial dysfunction in the context of VML injury, we sought to buffer ROS using an exogenous mitochondrially targeted peptide associated with improving redox balance, SS‐31 (also known as elamipretide or bendavia, and currently in clinical trials for various mitochondrial syndromes and myopathies). In our first trial of evaluation for a VML injury indication, SS‐31 was provided only during the first 2 weeks postinjury, with treatment beginning immediately postinjury. Strengthening our other mitochondrial approaches, SS‐31 resulted in the reduction of ROS emissions, and mitochondrial function was improved at 1 and 2 weeks post‐VML. Furthermore, even after SS‐31 was discontinued at 1 month postinjury (2 weeks of treatment, 2 weeks without) there was a remaining reduction in ROS emission.

### 3.1 Regenerative rehabilitation

Rehabilitation alone has shown limited efficacy for VML‐injured muscle (for a review, see Greising et al., 2019), in part owing to the limited oxidative plasticity of the muscle remaining. Thus, a combined approach that targets maladaptive plasticity with rehabilitation might be more effective. As a field, regenerative rehabilitation continues to grow. The foundation for regenerative rehabilitation is the combination of strategies from both physical rehabilitation and regenerative medicine fields, in efforts to restore function. We are currently working to develop formoterol as a regenerative treatment with rehabilitation. We expect that a combined approach with formoterol will continue to improve skeletal muscle quality and overall muscle function after VML injury. With a key tenet of regenerative rehabilitation that it improves the repair and regeneration of the tissue at both cellular and molecular levels, we sought to evaluate formoterol.

In our first attempt (McFaline‐Figueroa et al., 2025), we used two separate physical rehabilitation protocols, both with voluntary wheel running. As in our foundational studies, noted above, we began oral formoterol treatment immediately following VML injury, then we waited 1 week or 1 month before beginning the voluntary running. Running continued until 2 months post‐VML in both groups, and the daily running distance was similar and within normal ranges for both groups, at ∼3.4 km/day. Both muscle and metabolic function were improved with regenerative rehabilitation, especially in the experimental group that started 1 month post‐VML. The regenerative rehabilitation group demonstrated greater torque production per body mass, and using the CK clamp technique described above there was greater electron conductance. Directly comparing the regenerative rehabilitation approach with formoterol treatment alone, there was a ∼4.5% greater number of total myofibres. Combined with the noted increase in the number of myofibres with formoterol treatment in uninjured muscle noted above (Bijwadia et al., 2023), we now have increasing evidence to support *de novo* regeneration of fibres with formoterol treatment. However, this could also represent a preservation of fibres after injury. Notably, there are still aspects of this approach that need to be improved upon. In these studies, the formoterol treatment began before the rehabilitation, but continued in tandem. Many aspects of the timing and duration of each treatment have yet to identified. In our most recent attempt (Heo et al., 2025), we used a sequenced regenerative rehabilitation approach, again with voluntary running, but using the mitochondrially targeted peptide SS‐31, noted above. Initially, SS‐31 was delivered for the first 2 weeks post‐VML, then the rehabilitation started until 2 months post‐VML. Daily running distances were comparable between groups and similar to prior experimental groups. Our first three attempts at regenerative rehabilitation with a metabolic target show promise above pharmaceutical treatments alone.

## 4 IS TIMING EVERYTHING?

In both of our approaches to regenerative rehabilitation to date, we have delayed the start of rehabilitation by ≥1 week; however, there are still unanswered questions regarding the timing for rehabilitation alone and with regenerative approaches. We and others have begun various forms of the rehabilitation after VML injury as early as 3 days postinjury and as late as 1 month (Aurora et al., 2014; Basten et al., 2023; Habing et al., 2024; Hoffman et al., 2025; Hu et al., 2022; Johnson et al., 2024; McFaline‐Figueroa et al., 2025; Nicholson et al., 2024; Schifino et al., 2023; Southern et al., 2019; Ziemkiewicz et al., 2023). Across the field working to develop treatments for VML injury, rehabilitation has been the approach in which a delay between VML injury and beginning intervention is common. In part, this is straightforward experimentally, because there is no need for a second procedure as is common with implantable treatments. Although there are delays in initiating rehabilitation, both in our group and across the field, the motivation or mechanistic rational to support this delay is not always clear.

From the perspective of fibrotic tissue deposition, we recently moved into delaying rehabilitation until 2 weeks post‐VML (Nicholson et al., 2024). This 2 week delay aligned with the time when we identified the fibrotic scaring post‐VML to be maturing fully (Hoffman et al., 2022), in addition to representing a time when the VML‐induced secondary denervation was beginning (Hoffman et al., 2023) and mitochondrial ROS emission was expanding (Heo et al., 2025). Although currently not well understood mechanistically, within preclinical models of VML injury the 2 week postinjury time point continues to be identified as a pathophysiological tidemark. The 2 week time frame contrasts with known regenerative processes in skeletal muscle, in which the tissue remodelling and repair would be expected to dampen collagen deposition and increase myogenesis and angiogenesis. One could hypothesize that the early 2 week window postinjury could identify a transitional phase for = mitochondrial transcription and its regulation (Miller et al., 2025). Focusing on approaches likely to include regenerative rehabilitation, future work is needed to understand whether early pharmacological approaches can overcome the VML‐induced pathophysiology to support a more robust rehabilitative response.

Addressing the post‐VML muscle with regenerative treatments alone, primarily with a biomaterial‐based approach, has also been done in a delayed manner with varying degrees of success. Early preclinical work by others evaluated a cell‐loaded biomaterial in chronically injured VML, orthotopically implanting it 1 month post‐VML (Quarta et al., 2017). Although there was little impact on muscle function, this approach resulted in modest improvements in muscle structure. In another delayed approach, the design was initially to stabilize the VML injury with a provisional biomaterial as a void filler, then upon explant at 1 month post‐VML and a regenerative minced muscle graft was placed orthotopically in the muscle, as a definitive treatment for the muscle (Clark et al., 2024). Although there was limited functional improvement with this approach, design constraints on the material properties of the provisional biomaterial were identified. In our recent approach (McFaline‐Figueroa et al., 2025), we also used a delayed intervention of 1 month, injecting a poly(ethylene glycol) diacrylate based biomaterial loaded with formoterol for local delivery. Although the results were mixed, there emerged promising outcomes to continue to drive our innovation. Unfortunately, the formoterol‐loaded biomaterial had a negative impact on contractile function and the number of myofibres in comparison to leaving the injury non‐repaired. Fortunately, metabolic function was improved meaningfully. The formoterol‐loaded biomaterial resulted in the ability to remain more metabolically stable after injury, and electron conductance was ∼27% greater than in VML injuries left non‐repaired. Although it is likely that within the clinical population a surgically based intervention is expected to be delayed postinjury, only few preclinical studies have undertaken this type of experimental design, evaluating a delayed intervention. Whether the approach is regenerative, rehabilitative or both, understanding the muscle remaining and the limitations of the pathophysiology is imperative to the success of the intervention.

## 5 UNANSWERED QUESTIONS AND CLINICAL PERSPECTIVE

Although our ongoing innovation and that of others continues to move the field forwards, many questions remain. Within our work on metabolic treatments to date, all investigations have been in small animal models, but understanding how these treatments can address the muscle remaining in large animal models is needed. The scale of the VML injury size in our preclinical mouse model is only ∼0.4% of our large animal model (Greising et al., 2017). To model the limitations in the clinic better, scaling up to large animals is needed, with subsequent clinical trials. With a systemic pharmacological approach, there is also better modelling of the drug metabolism and dosing during scale‐up studies, to support future clinical studies.

Across the field, the investigation into any sex‐specific differences following VML injury has been limited. Even with our work, it has only been recently that we have evaluated both biological sexes (Heo et al., 2023; Hoffman et al., 2023). Metabolically, in male VML‐injured mice, we detected a decrease in pyruvate dehydrogenase activity, but pyruvate dehydrogenase activity was not decreased in female VML‐injured mice (Heo et al., 2023). We were surprised to determine that in female VML‐injured mice there were more fully innervated neuromuscular junctions and greater overlap of the pre‐ and post‐synaptic structures than in male VML‐injured mice (Hoffman et al., 2023). Together with recent findings from others that female VML‐injured rats have a greater regenerative response to a minced muscle graft treatment than male VML‐injured rats (Motherwell et al., 2025), it is reasonable to expect differences in treatment efficacy with biological sex. Our work to date has only evaluated male VML‐injured mice in the context of treatment with formoterol. As we move forwards, we are excited to investigate efficacy in both sexes.

The use of β_2_‐adrenergic receptor agonists has not been without question. Given that drugs such as formoterol are working through G‐protein‐stimulated cAMP production, there is a risk of increased heart rate or arrhythmias with systemic use. And although there have been reported potential limitations owing to systemic treatment, the evidence is mixed (Lama, 2002; Sears, 2002). Thus, it could be prudent to understand other delivery options or other similar therapeutics. In recent investigations using a rational drug design and a hybrid target‐based phenotypic screen, newer pharmaceuticals have been identified and developed that improve the therapeutic potential of β_2_‐adrenergic receptor agonists (Motso et al., 2025). Their work has identified a candidate drug that has moved into clinical trials (NCT05409924) for a metabolic indication, with investigations planned to understand whether it can enhance muscle growth and improve glucose homeostasis in type 2 diabetics. We view advancements in new drug development as additive to our efforts. As additional studies on formoterol, with and without rehabilitation, progress following VML, target‐based drugs (Motso et al., 2025) might emerge as strong candidates for clinical translation.

As studies move into the clinic, the design of trials will be a challenge. The injury pattern of VML clinically is highly heterogeneous, and clinical inclusion patterns could span very narrow to all inclusive. There is a clinical need to evaluate regenerative rehabilitative approaches, but the effects of these programmes probably need to be evaluated as independent variables. Although the use of FDA‐repurposed pharmaceuticals is a benefit to quick translation, it is possible that the individual target product profiles will need to be tailored to the phase of care. Early treatment approaches might need to have highly specific targets, such as metabolism or fibrotic development, and more complex combinations of biologics should probably be avoided. From a regulatory standpoint, treatment approaches delivered in tandem or in series are likely to have a simpler pathway to approval than treatments in combination. Yet complex options, such as biomaterial loaded with cargo such as pharmaceuticals, could reduce both pharmaceutical burden and cost, with an overall limit to potential off‐target systemic effects (e.g., arrhythmia, as noted above), but regulatory approval burdens would be increased. As more studies move across the translational pipeline into clinical trials, collaborative and multidisciplinary teams will need to work together to overcome trial design limitations.

## 6 CONCULSION

We recently posed the question, ‘When is the right time to initiate rehabilitation?’ (Greising & Call, 2024), to spark a conversation on timing for rehabilitation after non‐recoverable, traumatic skeletal muscle injuries, such as VML. Although this question is likely to remain for some time, our ongoing work seeks to define how the physiological limitations of remaining muscle can inform evidence‐based treatment windows, recognizing that early stabilization after acute injury might precede definitive care. Building on this foundation, here we have provided an up‐to‐date understanding of the pathophysiological limitations related to VML injury (Figure 1), in an effort to help explain the limited adaptive capacity of the muscle after VML injuries. Our studies have revealed key metabolic dysregulation after VML and shown that regenerative pharmacological approaches, such as formoterol, can partly correct these deficits, improving muscle mass, function and metabolism. However, because formoterol alone cannot fully restore pre‐injury muscle capacity, continued investigation into combined metabolic and regenerative strategies remains essential for advancing rehabilitation outcomes.

## AUTHOR CONTRIBUTIONS

Sarah M. Greising and Jarrod A. Call drafted the manuscript, approved the final version and agree to be accountable for all aspects of the work. All persons designated as authors qualify for authorship, and all those who qualify for authorship are listed.

## CONFLICT OF INTEREST

None declared.
